# Eco-Friendly Cork–Polyurethane Biocomposites for Enhanced Impact Performance: Experimental and Numerical Analysis

**DOI:** 10.3390/polym16070887

**Published:** 2024-03-24

**Authors:** Mateusz Dymek, Mariusz Ptak, Paweł Kaczyński, Fábio A. O. Fernandes, Ricardo J. Alves de Sousa, Gabriel F. Serra, Maria Kurańska

**Affiliations:** 1 Faculty of Mechanical Engineering, Wroclaw University of Science and Technology, Lukasiewicza 7/9, 50-371 Wroclaw, Poland; 2 TEMA—Centre for Mechanical Technology and Automation, Department of Mechanical Engineering, University of Aveiro, Campus de Santiago, 3810-193 Aveiro, Portugal; 3 LASI—Intelligent Systems Associate Laboratory, 4800-058 Guimaraes, Portugal; 4 Department of Chemistry and Technology of Polymers, Cracow University of Technology, Warszawska 24, 31-155 Cracow, Poland

**Keywords:** cork, composite, polyol, foam, impact, strain energy density, finite element analysis, sustainability

## Abstract

Cork composites are byproducts from wine stopper production, resulting from the agglomeration of cork granules with a thermoset resin. The resulting compound is a versatile and durable material with numerous industrial applications. Due to its unique properties, such as low-density, high-strength, excellent energy absorption, and good thermal and acoustic insulators, cork composites find room for application in demanding industries such as automotive, construction, and aerospace. However, agglomerated cork typically has a polyurethane matrix derived from petrochemical sources. This study focuses on developing eco-friendly porous polyurethane biocomposites manufactured with the used cooking oil polyol modified with cork. Since cork and polyurethane foam are typically used for impact shock absorption, the manufactured samples were subjected to impact loading. The assessment of crashworthiness is performed through 100 J impact tests. A finite element numerical model was developed to simulate the compression of these new composites under impact, and the model validation was performed. The highest specific absorbed energy was obtained for petrochemical polyol composites with the 3% addition of natural or modified cork. The research conducted in this study showcased the feasibility of substituting certain petrochemical components used for the synthesis of the polyurethane matrix with ecological waste vegetable oil components.

## 1. Introduction

Functional materials from renewable resources have gained significant attention in recent years due to the need for sustainable and eco-friendly materials. These materials are derived from natural resources, such as plants, algae and bacteria, which can be renewed or regenerated without depleting the Earth’s resources. Functional materials refer to those materials that possess specific chemical, physical, or biological properties that make them suitable for various applications [1,2]. These materials have unique properties such as high strength, flexibility, and durability, making them ideal for use in various fields such as energy, biotechnology, medicine, and electronics. Using cork composites can reduce the environmental impact of industries, helping tackle climate change [3]. Furthermore, cork composites are recyclable and biodegradable, making it an attractive option for companies looking to reduce their carbon footprint [4,5,6,7].

In order to investigate the mechanical behavior of cork composites under the impact, Gameiro et al. [8] conducted a study that compared the material’s response to quasi-static and dynamic compressions while examining the effects of different cork types, densities, moisture, cell structures, and strain rates. To analyze the energy absorption capacity, the authors compressed circular and square aluminum tubes filled with cork at different static and dynamic deformation rates. They used commercial finite element software to simulate the dynamic compression tests [9,10]. The results demonstrated excellent agreement with experimental tests, suggesting the possibility of predicting structures filled with cork under dynamic loads

Similarly, Fernandes et al. [11] conducted dynamic impact tests on cork agglomerates using a 5 kg projectile dropped from a 3 m height to investigate their dynamic compressive behavior and relaxation during unloading. The experimental results showed that all the samples exhibited high elastic recovery, exceeding 95% after the first impact, making the material promising for equipment subjected to repeated impact loading. Comparing the numerical results obtained with their constitutive model, the authors found that the loading and unloading measurements aligned closely with the experimental results.

Protective components require the careful consideration of material thickness to ensure optimal performance. A study by Gameiro et al. [12] analyzed the influence of thickness on the energy absorbed during dynamic compression. The authors found that increasing thickness lowered maximum force and deformation while absorbing 70–80% of impact energy. This highlights the cork’s excellent energy absorption capacity, regardless of the number of impacts [13,14]. Furthermore, researchers examined the versatility of cork composites by testing different agglomerates with varying properties. They found that less dense agglomerates had a lower stress plateau and stored lower energy levels per unit volume [15,16]. At the same time, higher granule content caused more significant mechanical damage, making the material’s performance more compromised in multiple impact situations [12,17,18]. This research aligns the works carried out by Gerometta and Karbowiak et al. [19,20].

Different research groups have increasingly explored replacing synthetic materials with natural ones [21,22]. In a study conducted by the group of Fernandes et al. [23], two cork agglomerates (with densities of 216 and 199 kg/m^3^), expanded cork (with a density of 159 kg/m^3^), EPS (with a density of 90 kg/m^3^), and EPP (with densities of 60 and 90 kg/m^3^) were tested for their suitability in motorcycle helmets, which are characterized by high impact energies. EPS outperformed cork-based materials in a single-impact scenario [23]. However, due to their elastic nature, the cork agglomerates demonstrated better results when exposed to multiple impacts, which are more likely to occur during a moped accident. Static tests indicated that synthetic materials could absorb more energy under low stresses, but natural materials exhibited a better performance and resistance in dynamic situations involving multiple impacts [23,24].

Moreover, cork’s unique properties have attracted attention in different industries. Sergi et al. [21] discussed cork’s compressive behavior and crashworthiness, underscoring its appeal in various industrial sectors. Morillas et al. [25] focused on characterizing natural cork for acoustic solutions, aligning with the growing demand for sustainable materials. Additionally, Gürgen and Sheikhi et al. [26,27,28] explored the use of shear thickening fluids in cork agglomerates, demonstrating the material’s adaptability to innovative technologies. According to Fernades et al. [11], cork is nearly isotropic due to the random orientation of its granules, resulting in a Poisson coefficient of practically zero. Therefore, cork is a suitable material for applications requiring high dimensional stability. The researchers examined four white cork agglomerates in their study, including two with the same density but different granulate sizes and one expanded agglomerate. Their findings indicated that the sample’s energy absorption capacity decreased to less than a quarter as the temperature increased from −30 °C to 100 °C. Moreover, the density of the sample influenced the energy absorbed as the temperature increased. These findings should be considered when designing new products made from this natural material.

More recently, Sergi et al. [29] tested the resistance of sandwich structures containing cork agglomerates as the core material. They found that a higher density resulted in greater resistance and less maximum displacement, and the cork agglomerates showed an excellent capacity for dimensional recovery after compression. Using cork as a core material in sandwich structures allows for an environmentally friendly solution, improving the damage tolerance while only increasing the weight by 20%. Finally, Ptak et al. [30] conducted dynamic tests on the cork composites containing graphene oxide and graphene nanoplates and found no differences in the energy absorbed compared to the same material without graphene.

The ongoing trend of producing environmentally friendly materials [31] coming from the re-utilizations of wastes, ultimately adding value to byproducts, is the main motivation for the authors to perform mechanical tests on newly introduced samples by Kurańska et al. [23]. Polyurethane materials obtained by mixing renewable raw materials such as petrochemical polyol (PU) and petrochemical polyol and bio-polyol (BPU) in a 1:1 weight ratio are becoming attractive in various industries, including construction [32,33,34,35,36]. The detailed chemical composition, dimensional stability, water absorption, brittleness, and compressive strength are presented in the paper [37]. The experiments presented in this manuscript aim to verify the capabilities of energy absorption in dynamic impacts and its reconstruction with numerical methods. The energy of the impact was established as 100 J; the impactor velocity was set as 4.8 m/s.

## 2. Materials and Methods

### 2.1. Samples Composition

Ten cork composites, one petrochemical polyol foam, and one polyurethane and bio-polyol foam were submitted for tests. Prepared polyurethane (PUR) foams were produced through a one-stage method utilizing two-component systems, where component A was referred to as the polyol premix, and component B was the isocyanate. The polyol premix comprises a combination of ingredients, including a polyol or a blend of a petrochemical polyol and a bio-polyol, a catalyst, a surfactant, a flame retardant, and water. The polyol premix was prepared in 500 mL polypropylene containers. Subsequently, the appropriate quantity of isocyanate was introduced into the premix container and immediately agitated using a mechanical stirrer at 1800 RPM for 7 s.

**Figure 1:**
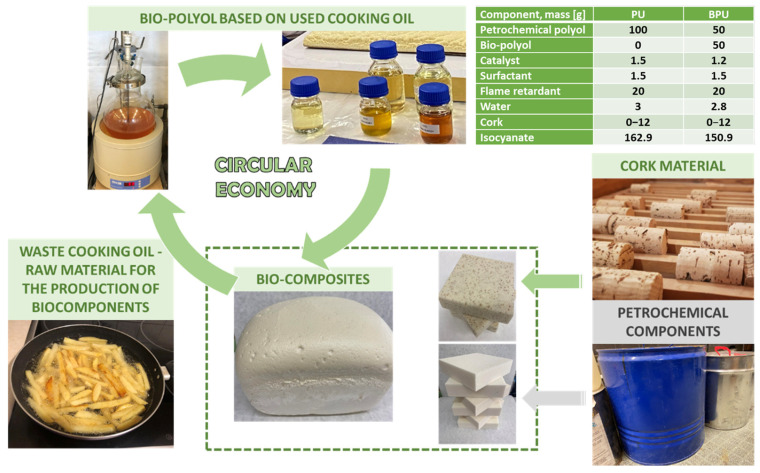
Composites manufacturing the graph depicting the petrochemical polyol foam, polyurethane, and bio-polyol foam used for impact tests.

The foams were produced through a single-step process utilizing two-component systems. Component A, referred to as the polyol premix, and component B, comprising an isocyanate, were employed. The polyol premix comprised a blend of either a petrochemical polyol or a combination of petrochemical polyol and bio-polyol, along with a catalyst, surfactant, flame retardant, and water. The polyol premix was prepared in polypropylene containers with a capacity of 500 mL. Subsequently, the appropriate quantity of isocyanate was added to the premix container and immediately stirred using a mechanical stirrer for 7 s. Composites were formulated with cork content ranging from 3% to 12% relative to the polyol mass of the PU system. Following mixing, the composition was poured into pre-prepared plastic molds, where it was expanded and cross-linked. Testing of the materials was conducted 24 h post-synthesis. Reference materials were synthesized using two distinct formulations: one solely based on petrochemical polyol (PU) and the other based on a 1:1 weight ratio blend of petrochemical polyol and bio-polyol (BPU). The isocyanate index for the synthesized materials was maintained at 1.1. The detailed manufacturing process was described in [37]. Figure 1 depicts the compositions of formulations per 100 g of the components.

Composites were formulated with A cork content ranging from 3% to 12% relative to the mass of the polyol in the petrochemical polyol system. Our goal was to obtain polyurethane composites with a maximum cork content. We analyzed the influence of processing possibilities (foaming process) with different cork contents and 12% was the maximum value that allowed the authors to obtain a reaction mixture with a viscosity acceptable from the processing point of view.

The composition was poured into pre-prepared plastic molds following this mixing process, allowing the material to expand and cross-link. The testing of these materials was conducted 24 h after their synthesis. As a point of reference, two sets of materials were synthesized. The first set was solely based on PU, while the second set incorporated a blend of petrochemical polyol and bio-polyol (BPU) in a 1:1 weight ratio. The specifics on the development and chemical composition is in the previously published paper by Kurańska et al. [37]. The materials are separated into the following groups:

1. One petrochemical polyol foam without cork (PU_0) and four petrochemical polyol with different amounts of modified cork (PU_CM_3, PU_CM_6, PU_CM_9, PU_CM_12);
2. Two foams of a petrochemical polyol with varying amounts of natural cork (PU_C_3, PU_C_6);
3. One petrochemical polyol and bio-polyol (mass ratio 1:1) foam without cork (BPU_0) and petrochemical polyol and bio-polyol (mass ratio 1:1) foams with varying amounts of modified cork (BPU_CM_3, BPU_CM_6, BPU_CM_9, BPU_CM_12).

**Figure 2:**
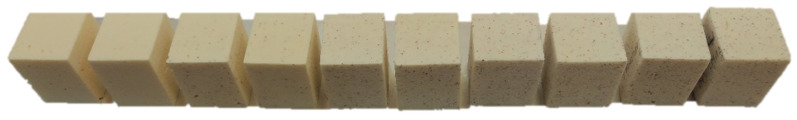
Sample images from the left: PU_CM_3, 6, 9, 12; PU_C_3, 6; BPU_CM_3, 6, 9, 12.

Table 1 presents the description of the composition of the samples and the nomenclature used in this work. All samples are 50 × 50 × 60 mm in size (width × depth × height). The manufactured samples, before the impact tests, are depicted in Figure 2.

**Figure 3:**
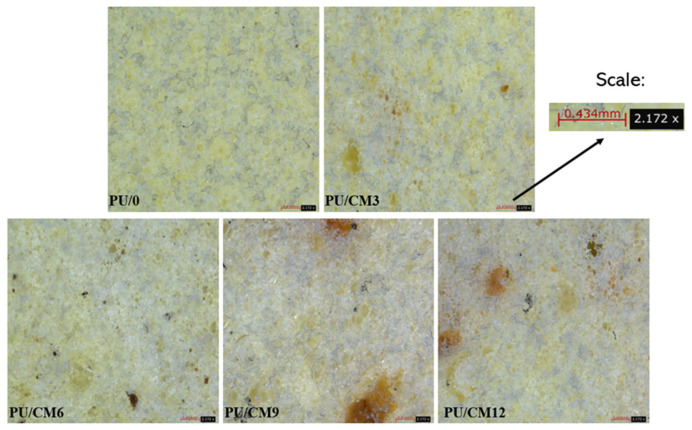

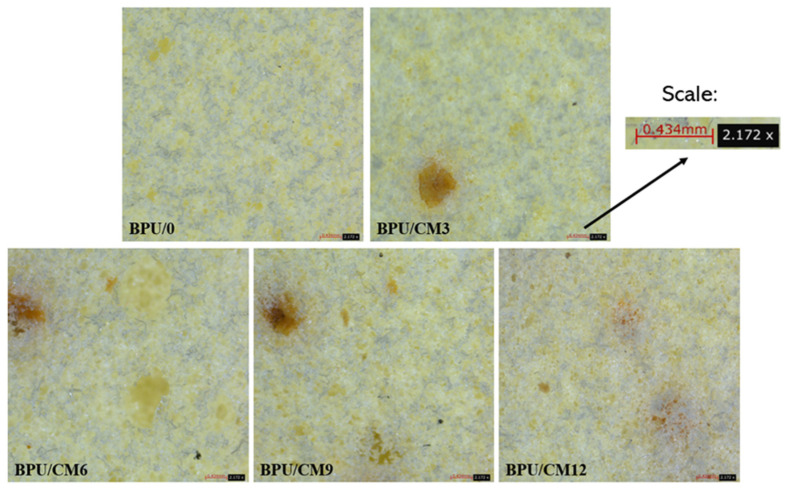
Samples of petrochemical polyol foams and bio-polyol foams—observed under a digital microscope.

The obtained foams were subjected to microscopic examination in order to compare the structure of each. The observations were conducted using a Leica Emspira 3 digital microscope (Figure 3). Upon observing the structure of rigid polyurethane foams with the naked eye and under a microscope, it can be noted that the addition of biopolyol in the form of used cooking oil did not significantly affect the appearance of the foams. The noticeable difference is a slightly more saturated color in the case of foams with biopolyol (BPU). Additionally, by comparing the samples with different percentages of cork material in the composition, it can be concluded that, as the proportion of cork material increases in the foam structure, more cork cells become visible.

**Figure 4:**
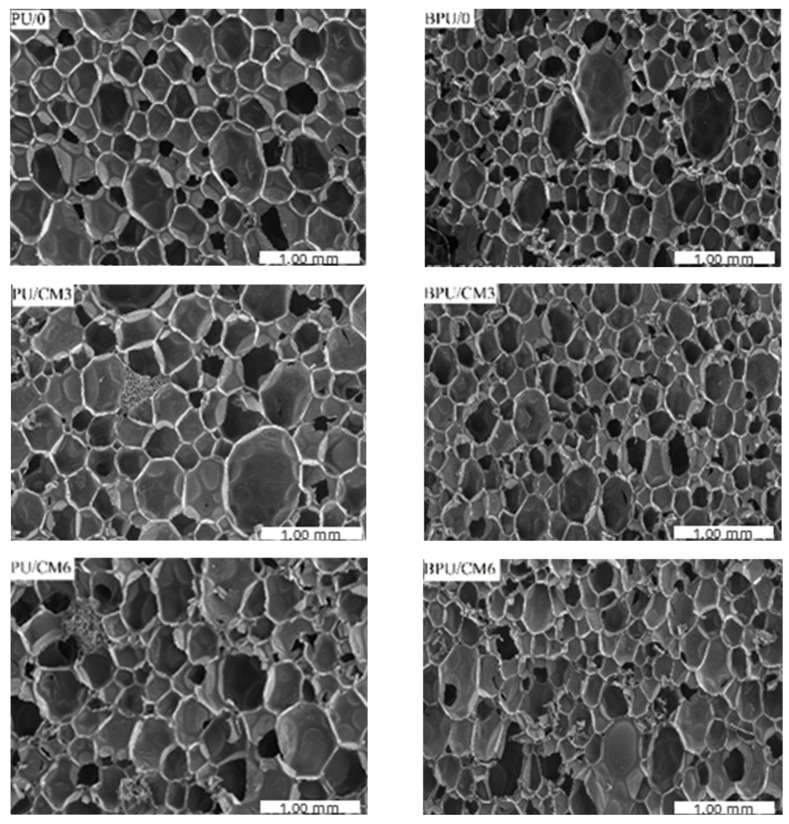

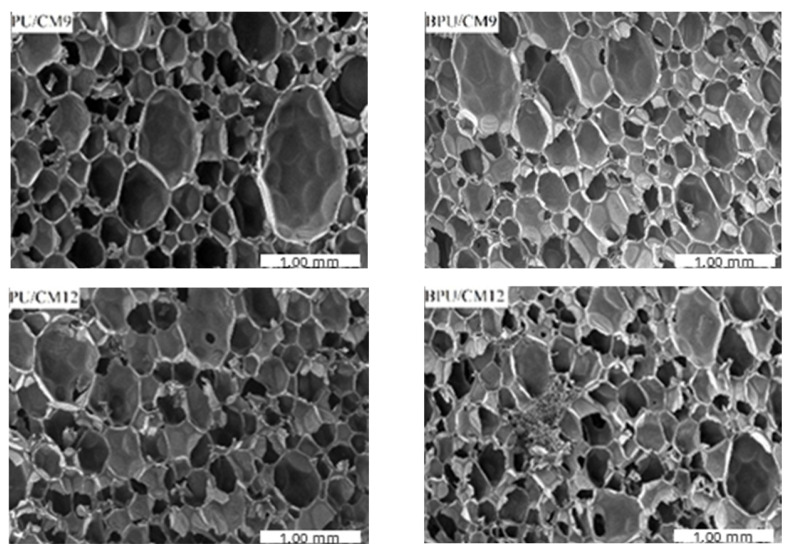
Scanning electron microscope (SEM) images of cellular structure of foams without (**left**) and with the biopolyol (**right**).

The SEM micrographs of the samples were taken to analyze the average cell surface area and anisotropy (Figure 4). Additionally, it was possible to determine the effect of the bio-filler content on the foams’ cellular structure. The cellular structure of PUR foams has a significant impact on their mechanical and thermal properties [37].

### 2.2. Physical Testing

**Figure 5:**
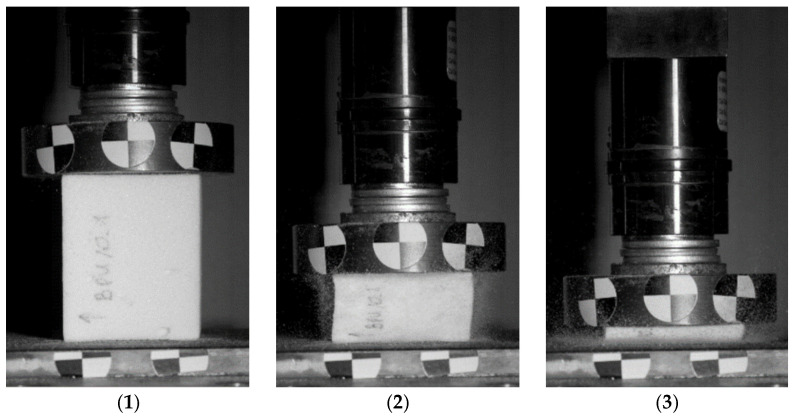

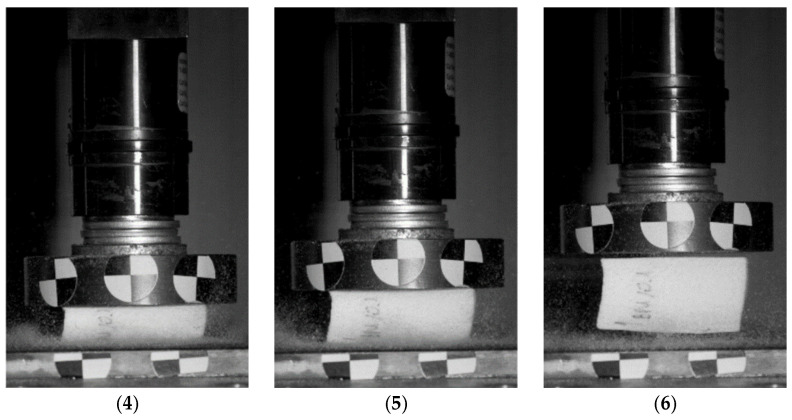
Experimental setup, cork sample BPU_0 at selected time intervals: (1–3) the compression process; (4–6) rebound of the impactor.

The dynamic impact tests with an initial impact velocity equal to 4.8 m/s were performed using the instrumented uniaxial drop-weight tower Instron Dynatup 9250HV. The experimental tests were conducted on all 12 cork composite samples using an 8.515 kg cylindrical steel impactor with a flat impact surface. The impact energy is set as 100 J. In all tests, the specimens were centered on the lower anvil. An ultra-high-speed camera Phantom V12 was used to record the impacts for visual analysis (Figure 5)—the footage is available at https://doi.org/10.5281/zenodo.8014308 (accessed on 22 March 2024). The machine setup with bumpers allowed the impactor to be moved to a maximum of 50 mm below the initial contact. Thus, the maximum experimental strain value obtained was 83%. The test stand enabled obtaining data such as the impactor’s displacement, velocity, and acceleration in time.

### 2.3. Numerical Testing

The samples were modelled as a nonlinear elastic material to replicate the experimental tests due to the small amount of permanent deformation observed minutes after the impact. Therefore, tested foams were modelled as a hyperelastic material, using the combination of Hyperfoam and Mullins effect material models available in the Abaqus CAE nonlinear material library.

The Hyperfoam material model is described by the foam elastic behavior based on the following strain energy function:

(1) $$\tilde{U}=\sum_{i=1}^{N}\frac{2\mu_{i}}{\alpha_{i}^{2}}\;\left[\lambda_{1}^{\alpha_{i}}+\lambda_{2}^{\alpha_{i}}+\lambda_{3}^{\alpha_{i}}-3+\frac{1}{\beta_{i}}\left({\left(J\right)}^{\alpha_{i}\beta_{i}}-1\right)\right]$$

where *N* is an integer (the polynomial order), J is the elastic volume ratio (J = $\lambda_{1},\lambda_{2}{,\lambda }_{3}$), $\lambda_{i}$ are the principal stretches, $\mu_{i}$ are the shear moduli, $\alpha_{i}$ and $\beta_{i}$ are the curve-fitting material parameters that are related to the material compressibility, where the initial bulk modulus, $K_{0}$, is given by the following expression [23]:

(2) $$K_{0}=\sum_{i=1}^{N}2\;\mu_{i}\left(\frac{1}{3}+\beta_{i}\right)$$

For each term in the energy function, the coefficient $\beta_{i}$ determines the degree of compressibility. The coefficient $\beta_{i}$ is related to the Poisson’s ratio, $\upsilon_{i}$, by the expressions:

(3) $$\beta_{i}=\frac{\upsilon_{i}}{1-2\upsilon_{i}}$$

(4) $$\upsilon_{i}=\frac{\beta_{i}}{1-2\beta_{i}}$$

Thus, if $\beta_{i}$ is the same for all terms, there is a single effective Poisson’s ratio, υ. The coefficients $\mu_{i}$ are related to the initial shear modulus, $\mu_{0}$, by:

(5) $$\mu_{0}=\sum_{i=1}^{N}\mu_{i}$$

The principal stretches $\lambda_{i}$ are related to the principal nominal strains $\varepsilon_{i}$ by:

(6) $$\lambda_{i}=1+\varepsilon_{i}$$

The Hyperfoam model was combined with Mullins effect properties that provide a mechanism to include permanent energy dissipation and stress softening effects in elastomeric foams. The Mullins effect is assumed to result from a microstructural evolution where the material expands, resulting from irreversible damage to its structure, at its relaxation after a load has been applied. Numerically, this phenomenon is usually seen when one wants to evaluate the material in its relaxation for validation or multi-impact testing.

With the Mullins effect, the stresses are computed by [23]:

(7) $$\sigma \left(\eta ,\lambda_{1}\right)=\eta \tilde{\sigma }\left(\lambda_{i}\right)$$

where $\tilde{\sigma }$ is the stress corresponding to the primary foam behaviour at the current deformation level $\lambda_{i}$. Hence, the stress is obtained by scaling the stress of the primary foam behavior with the damage variable η. The damage variable varies with the deformation according to:

(8) $$\eta =1-\frac{1}{r}erf\left(\frac{U^{max}-\tilde{U}}{m+\beta U^{max}}\right)$$

where $U^{max}$ is the maximum value of $\tilde{U}$ during the material deformation history, *r*, and β are dimensionless material parameters that control the amount of damage, and m controls whether the damage occurs at low strain levels, having the energy dimension. When $\tilde{U}$ = $U^{max}$, the equation stands as η = 1. However, when $\tilde{U}$ = 0, the damage variable η, attains its minimum value $\eta_{min}$. For all intermediate values of $\tilde{U}$, η varies monotonically between 1 and $\eta_{min}$. The recoverable part of the energy is obtained by subtracting the dissipated energy from the augmented energy as:

(9) $$U_{recoverable}=\eta \tilde{U}\left(\lambda_{i}\right)-\phi \left(\eta_{min}\right)$$

where the residual value of the augmented energy function $\phi \left(\eta_{min}\right)$, represents the energy dissipated due to damage in the material upon complete unloading. The damage energy accumulates with progressive deformation along the primary curve and remains constant during unloading. During unloading, the recoverable part of the strain energy is released. The latter becomes zero when the material point is completely unloaded. Upon further reloading from a completely unloaded state, the recoverable part of the strain energy increases from zero. When the maximum strain attained in the previous stage is again exceeded upon reloading, further accumulation of the damage energy occurs.

Table 2 represents the parameters introduced in the Abaqus 2021 software to characterize all samples where *N*, *m*, and *β* were introduced according to the literature [23]. Each sample is distinguished by its stress–strain curve obtained from physical experiments.

**Figure 6:**
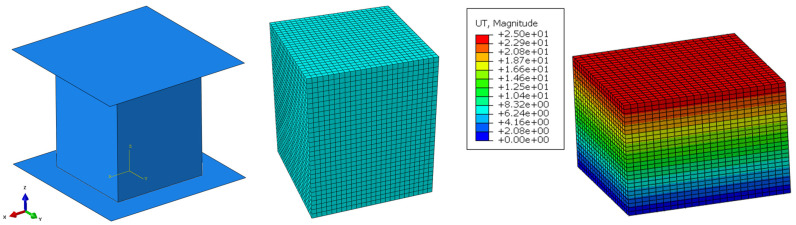
Simulation setup (visualization) (**left**); discretized model (**middle**); exemplary displacement (in millimeters) (**right**).

Two rigid analytical planes were modelled to represent the steel anvil and impactor to replicate the experimental dynamic tests in Abaqus. As in the experiments, the rigid impactor has one degree of freedom in the compression direction (*z* axis—Figure 2). A predefined field was added to represent the impact velocity of 4.8 m/s. The interaction between the samples and the rigid bodies was modelled with a surface-to-surface contact (explicit dynamic solver), using a friction coefficient of 0.2 and a “hard” contact pressure-overclosure. The friction coefficient used was investigated with other research of the authors [11]. The bottom plate was fully constrained to stay fixed. The deformable body was discretized using 32,805 hexahedral elements with reduce integration (Abaqus C3D8R—8-node linear brick, reduced integration with hourglass control). The typical displacement results are visible in Figure 6.

## 3. Results and Discussion

### 3.1. Physical Testing Results

**Figure 7:**
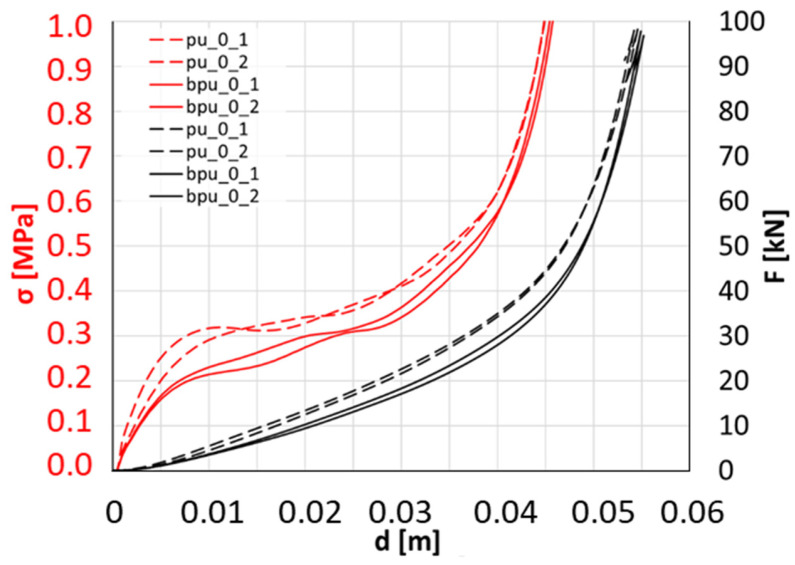
Comparison of compressive stress (red) and compressive force (black) of BPU and PU according to impactor displacement.

BPU sample obtained from petrochemical polyol and bio-polyol is characterized by dynamic testing with a 15% lower ability to absorb energy than PU sample obtained from petrochemical polyol (Figure 7). At the 45 mm impactor displacement after the initial contact, BPU absorbed 37.75 J, whereas PU absorbed 44.35 J. This phenomenon is observed due to the compressive stress reduction at the plateau region (0.1 < ε < 0.35), accordingly, σ_BPU_0_ = 0.283 MPa and σ_PU_0_ = 0.344 MPa.

**Figure 8:**
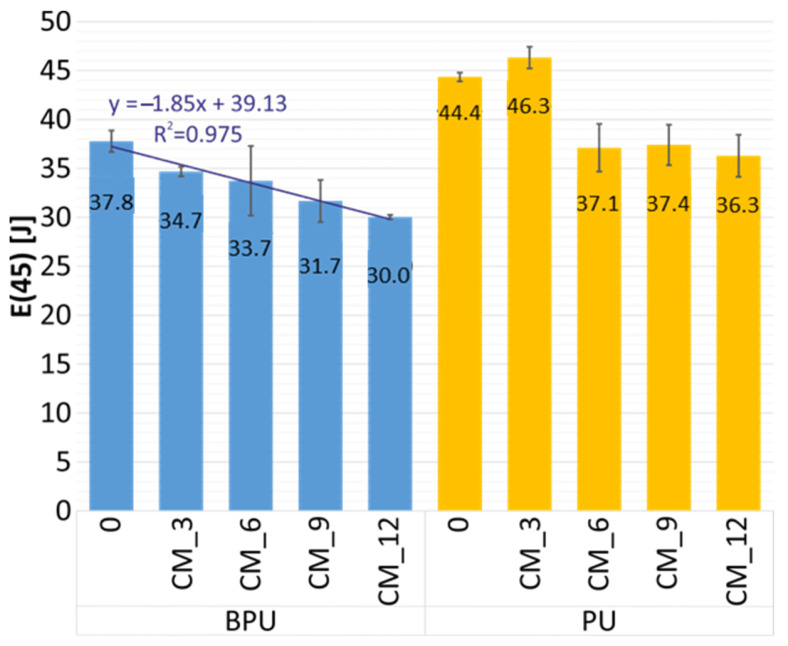
Influence of cork content on the energy absorption level at 45 mm impactor displacement after the initial contact.

Samples obtained by composing petrochemical polyol and bio-polyol (BPU_CM) absorb from 9% to 25% less energy (depending on modified cork content) than petrochemical polyol (PU_CM) samples (Figure 8). However, there is no visible trend between the cork content in samples and the level of energy absorption. The graph references the 45 mm impactor displacement after the initial contact. The most significant difference is noted between the samples with 3% modified cork content (Eavg_BPU_CM_3_/Eavg_PU_CM_3_ = 34.7/46.3 = 0.75). The lowest difference is observed between the samples with a 6% modified cork content (Eavg_BPU_CM_6_/Eavg_PU_CM_6_ = 33.7/37.1 = 0.90). The overall lower performance of BPU samples in terms of energy absorption is due to the lower content of hydroxyls in the bio-polyol concerning the petrochemical polyol. Due to the imperative of preserving an equivalent isocyanate index, incorporating bio-polyol results in a reduced quantity of isocyanate introduced into the PUR formulation, which, in turn, generates rigid segments within the PUR chains responsible for mechanical strength [37].

Interestingly, adding modified cork to samples restricts the capability to absorb impact energy. Inspecting petrochemical polyol samples with up to 3% cork content, there is no major influence on energy absorption (Figure 8). Additional amounts of cork decrease the impact of energy absorption by approximately 19% from 45.4 J (average value for PU_0 and PU_CM_3) to 36.9 J (average value for PU_CM_6, PU_CM_9, and PU_CM_12). Inspecting petrochemical polyol with bio-polyol samples, the increase in cork content influences the decline of energy absorption in a direct proportion. The formula can describe this phenomenon as E = −1.85·k + 39.13 (R^2^ = 0.975), where k is the percentage content of modified cork. This means that, with the 1% cork content increase, the ability to absorb energy declines by an average of 1.85 J, approximately 4.89% according to the base value of 37.8 J. Linear approximation is a helpful tool to optimize sample stiffness for a particular purpose. The cork’s impact on constraining the composites’ energy absorption capacity is closely tied to the system’s structural integrity. As illustrated in Kurańka’s prior investigation [37], incorporating cork filler dilutes the reaction system, thereby diminishing the reactivity, as measured through dielectric polarization. This reduced reactivity is further evident in the core temperature of the foams, with the presence of cork leading to lower maximum temperatures. Furthermore, introducing cork into the composite matrix induces an expansion in cell dimensions and a reduction in the apparent density of both BPU_CM and PU_CM samples. These changes significantly influence the structural integrity and consequently, impact the mechanical properties of the materials.

PU_CM_3 presents a higher energy absorption (at 45 mm impactor displacement) than PU_0. It is potentially related to an optimum value reached for this cork content, which, if higher, leads to lower energy absorption values. This is potentially the case since the energy absorption for PU_0 and PU_CM_3 is similar, and the same holds between PU_CM_6, PU_CM_9, and PU_CM_12, although this evolution is not linear. The fact that cork is a natural material and expanded cork increases the variability of this material (expanded microcells), and the variability associated with the granules processing probably leads to this nonlinear trend. Additionally, compared to the BPU, some compatibility issues between cork granules and PU might also justify it. Additionally, a higher cork content might weaken the PU/BPU matrix. In other words, the potential explanation for the lack of correlation between the cork content and energy absorption level in PU and the opposite in BPU, presenting a linear trend, are potentially justified by compatibility issues between the cork granules and the PU and vice versa with BPU.

The physical experiments resulted in calculating the stress–strain curves for each sample. These data are used as individual input in the FE analysis as well as a comparison for results.

### 3.2. Numerical Results and Comparison

**Figure 9:**
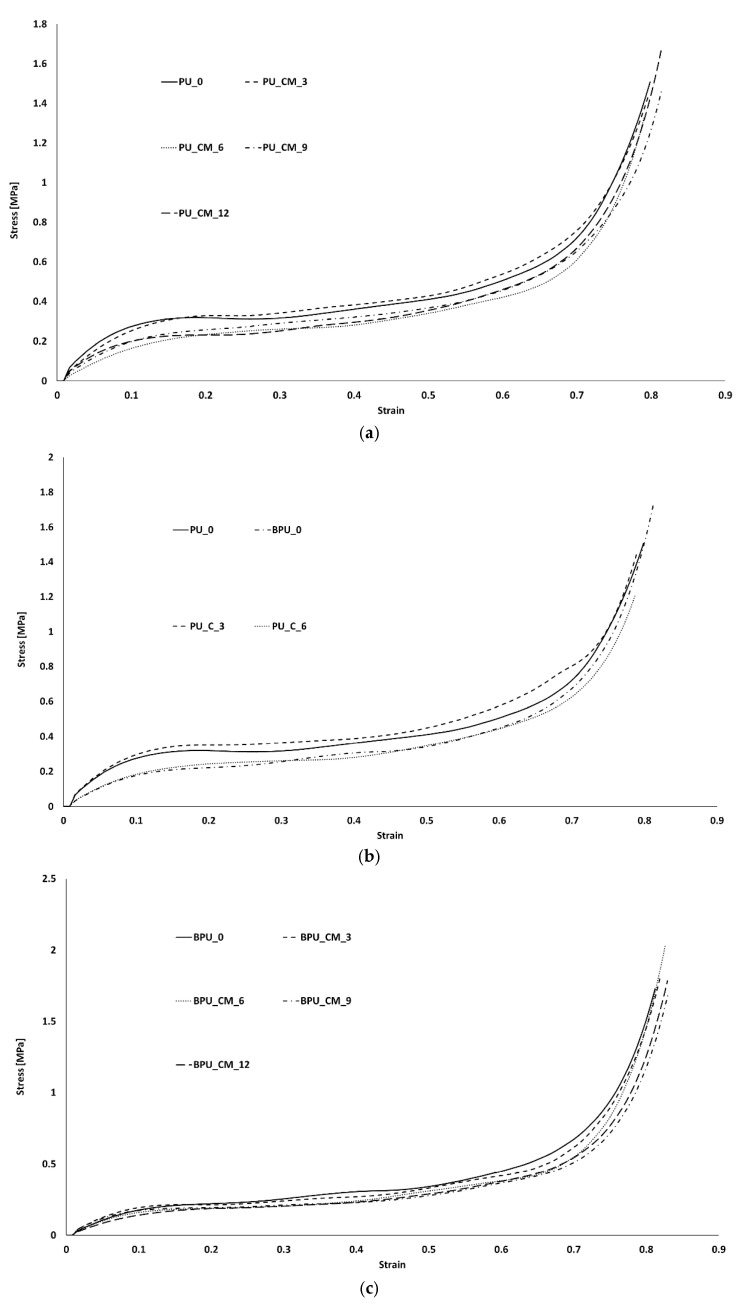
Uniaxial stress vs. uniaxial strain experimental curves: (**a**) PU_0, PU_CM_3, PU_CM_6, PU_CM_9, and PU_CM_12; (**b**) PU_0, BPU_0, PU_C_3, and PU_C_6; (**c**) BPU_0, BPU_CM_3, BPU_CM_6, BPU_CM_9, and BPU_CM_12.

**Figure 10:**
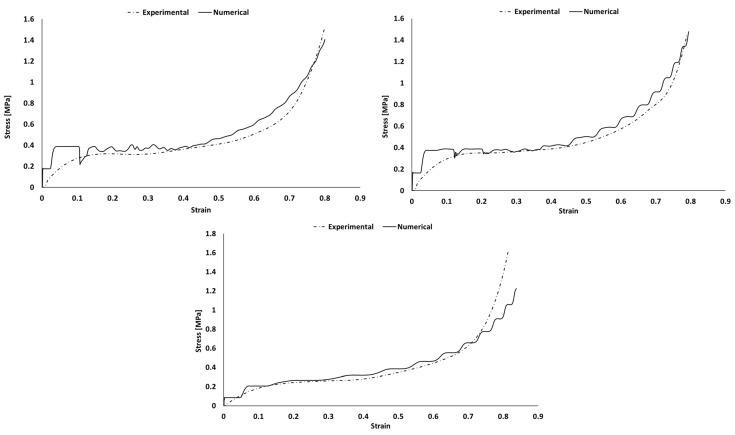
Experimental and numerical stress vs. strain of PU samples with and without the addition of natural cork: PU_0 (**top left**); PU_C_3 (**top right**); and PU_C_6 (**bottom**).

**Figure 11:**
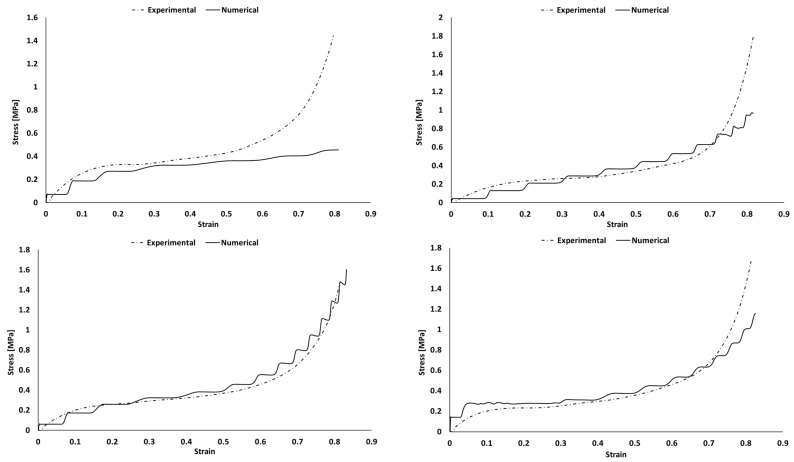
Experimental and numerical stress vs. strain of PU samples with the addition of modified cork: PU_CM_3 (**top left**); PU_CM_6 (**top right**); PU_CM_9 (**bottom left**); and PU_CM_12 (**bottom right**).

**Figure 12:**
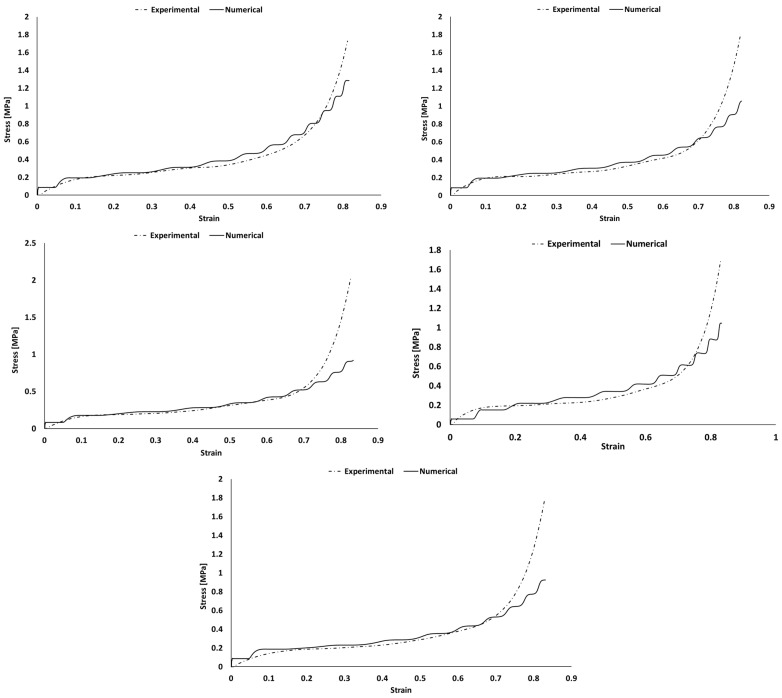
Experimental and numerical stress vs. strain of BPU samples with and without the addition of modified cork: BPU_0 (**top left**); BPU_CM_3 (**top right**); BPU_CM_6 (**middle left**); BPU_CM_9 (**middle right**); and BPU_CM_12 (**bottom**).

Each sample was simulated separately with Abaqus explicit solver. The comparison of experimental and numerical stress–strain curves is visible in Figure 9, Figure 10, Figure 11 and Figure 12). Depending on the sample, deviations are observed mostly during the densification stage. Although most of the experiments matched the simulations, there is a common discrepancy at high strains.

The Hyperfoam model was combined with the Mullins effect, providing a mechanism to include permanent energy dissipation and stress-softening effects in elastomeric foams. Numerically, this phenomenon is usually seen when one wants to evaluate the material in its relaxation for validation or multi-impact testing. Nevertheless, in this case, it helped to obtain a stable response, converging to the desired response of the experimental strain–strain curves. However, in some cases, it might be responsible for creating an early and unrealistic stress softening during loading.

In both simulation and experiments, the composites’ applicability is clear based on their mechanical response and performance. These present typical S-shaped stress–strain curves, which indicate their adequacy for crashworthiness and applications with the need for impact energy-absorbing materials/structures. Once the goal is to maximize the energy absorption, the mechanical response of each material should have a long plateau with moderate stress values, reaching densification for high strains.

The numerical curves depicting the behavior of pure PU samples and those incorporating natural cork (Figure 10) closely followed the experimental curves, with only a minor discrepancy observed in the PU_C_6 sample during the densification phase. The presence of a “staircase effect” in the numerical curves across all simulations may be attributed to numerical instability arising from contact between surfaces. Despite their somewhat irregular appearance, these curves effectively replicate the material behavior observed in experimental tests, yielding satisfactory results. Notably, when examining the numerical curves for PU samples containing modified cork (Figure 11), a more significant deviation from the experimental results was evident, particularly as these materials approached the densification phase. This discrepancy was particularly pronounced in the case of the PU_CM_3 sample, which failed to replicate this final stage of material behavior accurately. Nevertheless, it is important to highlight that the performance of this PU-modified cork composite within the linear elastic and plateau regions remains adequate for validating their suitability in energy-absorbing applications. These regions are pivotal in determining the material’s effectiveness for such purposes. Figure 12 shows that the BPU samples with modified cork present a shorter linear elastic region and a lower plateau, indicating a decreased resistance to impacts. This leads to smoother numerical curves and ultimately reflects a poor performance in terms of energy absorption.

### 3.3. Specific Absorbed Energy Profile

A global analysis of all composite’s performance was performed at this point. The goal is to maximize energy absorption. The mechanical response of each material should have a long plateau with moderate stress values, reaching densification for high strain values. The experimental strain energy per volume data was analyzed for an 80% strain level (at 0.80 strain value, the machine bumpers limited the impactor displacement to prevent the load cell damage), comparing the energy levels of each material.

**Figure 13:**
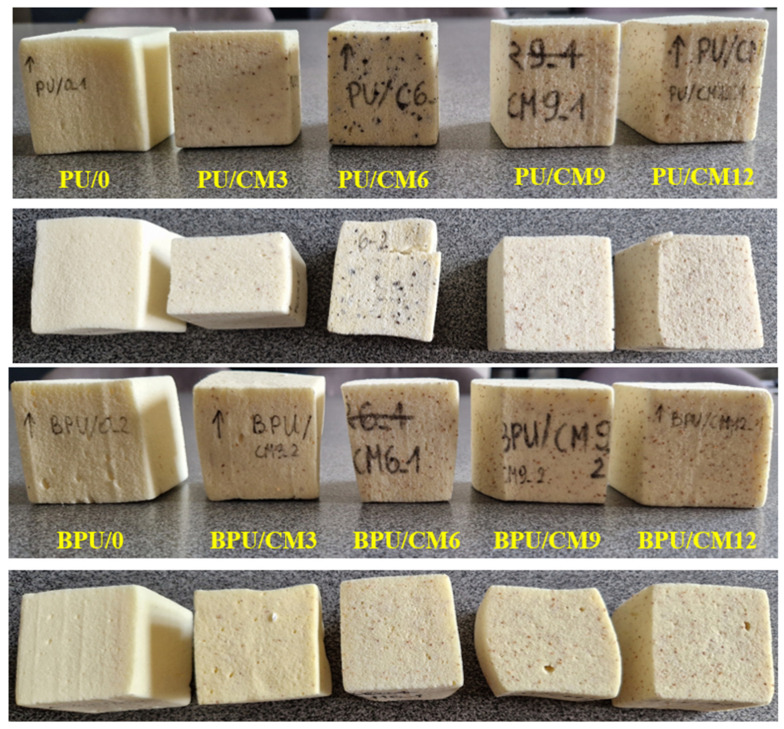
The specimens after impact tests: frontal and top views.

Analyzing Table 3, it is possible to notice that between PU samples, the PU_0, PU_C_3, PU_CM_3 and, respectively, BPU_0 obtained the highest values in terms of absorbed energy, confirming that these are the materials with the best performance in terms of energy absorption. Figure 13 depicts the samples after the impact loading.

## 4. Conclusions

In this study, new environmentally friendly composites were tested. Cork composites were developed, and samples were subjected to dynamic impact loading. The impact testing has shown that the petrochemical polyol foams and petrochemical polyol with bio-polyol (mass ratio 1:1) foams differ significantly with different modified or natural cork content. The testing setup and numerical simulations allowed the authors to obtain stress–strain data with energy absorption levels. It is observed that there is no noticeable relationship between the cork content and energy absorption level for PU samples. However, there seems to be a strong correlation between the cork content and energy absorption for BPU samples. The limitation of this study is that the samples were tested in one loading condition. Considering the literature review and the experience gathered from previous studies, we can assume that the strain rate will influence the results. Future studies should aim to investigate the sample behavior in different loading conditions and define the strain rate dependency.

The level of energy absorption is significantly lower than for cork agglomerates, yet the specimen mass is also lower. This leads to the conclusion that it will be challenging to introduce presented composites in energy–absorption structures subjected to high-energy impacts. However, one potential opportunity is the development of lightweight energy-absorbing layers for various technical purposes where low energy impacts are prevalent. These could include applications in automotive components, packaging materials, or sports equipment, where the demand for materials capable of absorbing and dissipating energy in low-energy impact situations is paramount. Cork material ability to withstand repeated impacts makes it a promising candidate for applications requiring durability under cyclic loading conditions. Overall, natural cellular materials have the potential to be used in developing advanced core materials and composites, with excellent mechanical resistance and outstanding crashworthiness properties competing against synthetic-based solutions, especially in situations that involve multiple impacts.

## Figures and Tables

**Table 1:** Cork composite samples composition and the nomenclature used.

Description	Cork Content	Nomenclature
Petrochemical polyol foam	-	PU_0
Petrochemical polyol foam	3% modified cork	PU_CM_3
Petrochemical polyol foam	6% modified cork	PU_CM_6
Petrochemical polyol foam	9% modified cork	PU_CM_9
Petrochemical polyol foam	12% modified cork	PU_CM_12
Petrochemical polyol foam	3% natural cork	PU_C_3
Petrochemical polyol foam	6% natural cork	PU_C_6
Petrochemical polyol and bio-polyol foam	-	BPU_0
Petrochemical polyol and bio-polyol foam	3% modified cork	BPU_CM_3
Petrochemical polyol and bio-polyol foam	6% modified cork	BPU_CM_6
Petrochemical polyol and bio-polyol foam	9% modified cork	BPU_CM_9
Petrochemical polyol and bio-polyol foam	12% modified cork	BPU_CM_12

**Table 2:** Model of material for cork samples.

ρ [kg/m^3^]	ν	N	r	m	β
90	0	3.0	1.1	0.5	0.1

**Table 3:** Energy absorption of all tested samples.

Samples	Strain Energy Density Up to 0.80 Strain Value (kJ/m^3^)
PU_0	360.36
PU_CM_3	369.98
PU_CM_6	293.14
PU_CM_9	311.46
PU_CM_12	309.68
PU_C_3	391.69
PU_C_6	297.33
BPU_0	306.56
BPU_CM_3	290.80
BPU_CM_6	263.29
BPU_CM_9	246.68
BPU_CM_12	250.53

## Data Availability

https://doi.org/10.5281/zenodo.8014308 (accessed on 22 March 2024).
